# Psychosocial Aspects of Rheumatic Disease Management: Addressing Mental Health and Well-Being

**DOI:** 10.7759/cureus.49267

**Published:** 2023-11-22

**Authors:** Syed Faqeer Hussain Bokhari, Aqsa Mushtaq

**Affiliations:** 1 Surgery, King Edward Medical University, Lahore, PAK; 2 Psychology, Quaid-i-Azam University, Islamabad, PAK

**Keywords:** patient-centered care, multidisciplinary approach, treatment adherence, coping strategies, chronic pain, emotional burden, well-being, mental health, rheumatic disease, psychosocial

## Abstract

Rheumatic disease imposes a substantial emotional burden on individuals, leading to chronic pain, discomfort, anxiety, and depression. Coping with unpredictability and physical limitations can create a detrimental feedback loop, exacerbating mental and physical symptoms. Poor mental health can hinder treatment adherence and worsen disease progression. Addressing the emotional impact of rheumatic disease is crucial for comprehensive management. Healthcare providers play a critical role in recognizing psychosocial concerns through attentive listening and standardized screenings. Open communication and a collaborative approach lead to more effective care. Support systems involving family, friends, and support groups provide emotional help and reduce isolation. Coping strategies and self-management techniques empower patients to navigate their conditions. The stigma associated with mental health is a challenge that requires education, awareness, and patient advocacy. A multidisciplinary approach integrating mental health services is pivotal for addressing the psychosocial aspects of rheumatic disease, offering a holistic perspective. Ongoing research explores the interplay between mental health and physical symptoms, aiming for innovative therapies and improved patient care. Collaborative care models and patient advocacy are essential to reducing barriers and improving patient outcomes. The future of rheumatic disease management lies in a patient-centered, multidisciplinary approach that addresses both physical and mental aspects.

## Editorial

Psychological impact of rheumatic diseases

Rheumatic diseases refer to a group of conditions characterized by inflammation, pain, and stiffness in the joints, muscles, or connective tissues. These diseases are often autoimmune in nature, meaning that the immune system mistakenly attacks healthy tissues. Common rheumatic diseases include rheumatoid arthritis, systemic lupus erythematosus, osteoarthritis, gout, ankylosing spondylitis, and juvenile idiopathic arthritis. Symptoms vary but often include joint pain, swelling, and reduced range of motion. Diagnosis typically involves a combination of medical history, physical examination, and, in some cases, imaging and laboratory tests. Treatment aims to manage symptoms, reduce inflammation, and improve the overall quality of life. Medications, physical therapy, and lifestyle modifications are common components of rheumatic disease management. Early detection and appropriate management are crucial for preventing long-term joint damage and complications.

Rheumatic diseases have a significant psychological impact on patients. They often bring about chronic pain and discomfort, leading to frustration, anxiety, and even depression in those affected. Coping with the uncertainty of flare-ups and unpredictable symptom changes can be emotionally taxing, as individuals may constantly fear the next episode. The impact on daily life, including limitations in physical activities and mobility, can lead to feelings of helplessness and a loss of independence [1].

The chronic nature of these conditions often results in emotional distress, contributing to anxiety and depression. This creates a detrimental feedback loop where mental health issues exacerbate physical symptoms, further intensifying the emotional burden. Moreover, poor mental health can hinder an individual's adherence to treatment plans, potentially leading to worsened disease progression. High stress levels, commonly associated with the management of rheumatic disease, can exacerbate inflammation and other physical symptoms, compounding the challenges individuals face. Fatigue, which is common in these conditions, can reduce motivation, further affecting emotional health. Functional limitations resulting from rheumatic disease may lead to frustration as individuals struggle to perform everyday tasks and engage in social activities.

Moreover, the quality of life can be compromised as individuals face challenges in maintaining relationships, pursuing their interests, and enjoying life to the fullest. Therefore, addressing the emotional impact of rheumatic disease is crucial in the comprehensive management of these conditions. Providing psychological support, pain management strategies, and helping individuals adapt to their limitations can significantly improve their mental health and overall quality of life.

Psychosocial needs of individuals with rheumatic disease

Healthcare providers play a critical role in recognizing and addressing psychosocial concerns in patients with rheumatic disease. This responsibility involves actively engaging with patients by listening attentively, observing emotional cues, and inquiring about their well-being during medical appointments. The ability to identify anxiety, depression, and other psychosocial issues early is essential as it allows for timely intervention and support. Comprehensive assessments in healthcare encompass both physical and mental health evaluations. The use of standardized mental health screenings can be particularly valuable in identifying issues that patients may be hesitant to openly discuss. Combining information from medical history, physical examinations, and mental health assessments provides a holistic perspective on the patient's overall well-being.

Open and honest communication between patients and healthcare providers is crucial in this process. Patients should feel comfortable sharing their psychosocial concerns, and a collaborative approach ensures that treatment plans consider both physical and mental health aspects. This collaborative approach ultimately leads to more effective and holistic care for individuals with rheumatic disease. Recognizing and addressing psychosocial needs in rheumatic disease management has significant benefits, including better patient outcomes, an improved quality of life, and a more comprehensive and patient-centered approach to healthcare [2].

Support systems and coping mechanisms

Individuals dealing with rheumatic disease benefit greatly from the support network of family and friends. This network plays a crucial role in providing emotional support and reducing feelings of isolation and depression. The understanding and empathy of loved ones significantly contribute to a better quality of life for patients by creating a nurturing environment. Coping strategies are often necessary for managing pain and the emotional challenges associated with rheumatic disease. Self-management techniques, such as maintaining a balanced lifestyle, engaging in physical activity, and employing relaxation methods, empower patients to navigate their conditions effectively. Support groups offer a valuable platform for patients to connect with others who face similar challenges. Within these groups, sharing experiences and advice can be both empowering and informative, fostering a sense of community. Online communities provide additional convenience, particularly for individuals with limited physical mobility. They offer a sense of belonging and connectedness, enhancing the overall support system [3].

Stigma and advocacy

Individuals living with rheumatic disease often confront the stigma associated with their mental health. This stigma can result in feelings of isolation, fear, and reluctance to seek help for their mental well-being. Addressing this issue requires a multifaceted approach, including education, raising awareness, and fostering a supportive environment to eliminate the associated stigma. Patient advocates play a crucial role in challenging stereotypes and advocating for the mental health of those with rheumatic disease. They leverage their personal experiences to promote understanding and drive change in the perception of mental health in this context. Collaborating with patient advocacy groups can magnify the impact of their efforts, creating a collective voice for change. Healthcare providers have a responsibility to engage in open conversations about the mental health needs of patients with rheumatic disease. Such dialogues ensure that mental health receives the attention it deserves in the development of treatment plans. Moreover, training healthcare professionals to address mental health concerns in this population is vital to providing comprehensive care. These actions aim to create a more supportive and inclusive environment for these patients [4].

Multidisciplinary approach

A multidisciplinary approach to care is pivotal in effectively managing the psychosocial aspects of rheumatic disease and providing holistic support for patients [5]. Collaborative care models unite a spectrum of healthcare professionals to address both physical and mental health requirements. Rheumatologists focus on diagnosing and treating the physical aspects of the disease, while psychologists and social workers offer vital mental and emotional support. This collaborative teamwork ensures a comprehensive approach to patient care, addressing both physical and psychological well-being. Additionally, examining case studies can offer valuable insights into successful strategies for psychosocial support. These cases may highlight the positive impact of collaborative care, coping mechanisms, and support systems, ultimately guiding healthcare providers in delivering more effective care.

Furthermore, the integration of mental health services within rheumatology clinics is indispensable. This integration reduces the stigma associated with seeking separate mental health care, making it more accessible and less daunting for patients. By offering comprehensive care in one location, this approach enhances access to services and, consequently, improves patient outcomes. Overall, a multidisciplinary approach, illustrated by case studies and bolstered by the integration of mental health services, presents a promising avenue for addressing the intricate psychosocial aspects of rheumatic disease management.

Future directions and challenges

As we look to the future of rheumatic disease management, it is crucial to emphasize ongoing research in psychosocial aspects. Emerging studies focus on the psychological well-being of patients, exploring how their emotional state impacts disease outcomes. These investigations aim to uncover the complex interplay between mental health and physical symptoms, offering insights into potential interventions and improved patient care. These studies may involve innovative therapies, holistic approaches, and the integration of mental health services within rheumatology clinics.

Providing comprehensive psychosocial care for individuals with rheumatic diseases remains challenging. Obstacles include restricted access to mental health services, the stigma associated with mental health issues, and the necessity for healthcare providers to adeptly address psychosocial concerns. It is crucial to acknowledge these challenges and work toward overcoming them to ensure that patients receive holistic care that addresses their mental and emotional needs. To address these challenges, potential solutions lie in collaborative care models that involve rheumatologists, psychologists, and social workers. These multidisciplinary teams can work together to provide holistic care that considers both the physical and mental aspects of rheumatic disease. Additionally, patient advocacy plays a vital role in raising awareness and reducing the stigma associated with mental health issues in the context of chronic illness. The path forward involves greater integration of mental health services within rheumatology clinics, emphasizing the importance of a patient-centered, multidisciplinary approach to care.
